# The digital heartbeat: a qualitative descriptive study on women's views on preventing cardiovascular disease in primary care

**DOI:** 10.1093/fampra/cmag041

**Published:** 2026-06-19

**Authors:** Ilhem Chaima Bousbiat, Samira Abbasgholizadeh-Rahimi, Roland Grad, Charo Rodríguez

**Affiliations:** Department of Family Medicine, School of Medicine, Faculty of Medicine and Health Sciences, McGill University, Montreal, QC H3S 1Z1, Canada; Department of Family Medicine, School of Medicine, Faculty of Medicine and Health Sciences, McGill University, Montreal, QC H3S 1Z1, Canada; Lady Davis Institute for Medical Research, Montreal, QC H3T 1E2, Canada; Mila-Quebec AI Institute, Montreal, QC H2S 3H1, Canada; Department of Family Medicine, School of Medicine, Faculty of Medicine and Health Sciences, McGill University, Montreal, QC H3S 1Z1, Canada; Lady Davis Institute for Medical Research, Montreal, QC H3T 1E2, Canada; Department of Family Medicine, School of Medicine, Faculty of Medicine and Health Sciences, McGill University, Montreal, QC H3S 1Z1, Canada; Institute of Health Sciences Education, Faculty of Medicine and Health Sciences, McGill University, Montreal, QC H3A 1A3, Canada

**Keywords:** women’s health, cardiovascular disease, primary prevention, AI in primary care, patient-centered care

## Abstract

**Background:**

This empirical study aims to explore women’s perspectives on cardiovascular disease and the use of digital health interventions (DHIs) for their primary prevention and to gather insights on essential features for developing artificial intelligent–enabled technologies.

**Methods:**

Adopting a qualitative descriptive research design, we conducted 15 semi-structured, in-depth interviews via Zoom with women at higher risk for cardiovascular disease. Participants were women over 40 years old, residing in Quebec, with at least one cardiovascular disease risk factor, and proficient in English. Recruitment was from a McGill University-affiliated clinic. An inductive thematic analysis approach was used for data analysis.

**Results:**

Five major themes were identified: (i) understanding cardiovascular disease in a variety of ways, (ii) barriers and challenges to preventing cardiovascular disease in women, (iii) women taking charge of their cardiovascular well-being, (iv) mixed perspectives regarding artificial intelligent–enabled technologies for cardiovascular disease prevention such as Xi-Care, and (v) range of suggestions for the format and design of a prospective artificial intelligent–enabled technologies.

**Conclusions:**

Despite the prevalence of cardiovascular disease, there is a significant knowledge gap among women regarding the chronic nature and manifestations of these diseases. Artificial intelligent–enabled technologies like Xi-Care, with the potential for customization and interactive engagement, could enhance the primary prevention of cardiovascular disease in women, providing valuable insights for the subsequent phases of the project leading to Xi-Care’s development.

Key messages

- **Limited awareness and knowledge gaps**. Women have limited understanding of cardiovascular diseases, highlighting the need for education.
- **Interest in personalized AI-enabled digital tools in women's preventive care**. Women show a strong interest in AI-enabled digital tools like Xi-Care that have the potential to offer personalized and interactive support for cardiovascular health.
- **Importance of tailored features for effective prevention**. Effective AI prevention tools must include customizable features such as health tracking, educational modules, and culturally tailored content.

## Introduction

Cardiovascular diseases (CVDs) affecting the heart and blood vessels have increased globally over the past three decades [1]. In 2019, CVD was responsible for 32% of all worldwide deaths, surpassing COVID-19 deaths by more than 2-fold in 2020 [2]. Historically associated with men, there are many misconceptions about the prevalence and severity of CVD in women despite it being the leading cause of death among women [3]. In Canada, CVD claims one life every 20 minutes, five times more than breast cancer [4].

Fortunately, CVD prevention is transforming through digital health interventions (DHIs), changing healthcare approaches to disease prevention while enhancing higher-quality care [5, 6]. Primary preventive strategies involve lifestyle changes, such as increased physical activity and dietary improvements, impacting cardiovascular risk factors (e.g. hypertension) [7–9]. A notable category of DHIs is mobile health technology (mHealth), which employs mobile applications to facilitate lifestyle modifications and improve adherence [10, 11]. In addition, recent evidence suggests that DHIs offer a compelling opportunity for CVD primary prevention. For example, cardiovascular screening, advancing precise cardiovascular care, and sex-tailored risk prediction models [12, 13].

However, existing healthcare inequalities, particularly for women, are reflected in AI training data, leading to biased predictions that disproportionately affect women and underrepresented minorities [12–14]. Underrepresentation and systemic data differences make AI models less accurate for these groups. For example, in cardiovascular screening, AI-enabled tools trained mainly on male data may miss symptoms or risk factors more common in women [12], resulting in misdiagnosis or delayed treatment and worsening health disparities. Such biases impact clinical decision-making by distorting the information clinicians rely on for diagnosis and treatment planning. If an AI model underestimates a woman’s cardiovascular risk, clinicians might not recommend preventive measures, negatively affecting patient outcomes [14].

In this context, the pertinence of conceiving and developing an AI-enabled tool, known as Xi-Care, emerged. Xi-Care aims to provide personalized recommendations for preventing CVD, guiding women through personalized health decisions and lifestyle adjustments. This study represents the initial stage of a comprehensive project funded by Canadian Institute of Health Research (CIHR), led by our team (S.R.). The following research questions drove our research work:

1. What are women's perspectives on cardiovascular disease and the use of DHIs for their primary prevention?
2. What are women's suggestions regarding developing an AI-enabled tools for cardiovascular disease prevention in primary care?

## Methods

### Study design and setting

We conducted a qualitative descriptive study at a primary care clinic linked to McGill University in Montreal, Canada. We chose this setting due to its affiliation with the research team's academic unit and its accessibility for participant recruitment and data collection [15]. We selected a qualitative descriptive approach because it is a generic qualitative research design that enables a comprehensive exploration of women's viewpoints by examining their personal experiences while accounting for the contextual factors that shape their perspectives [16].

### Participant selection

Following a purposive sampling approach, participants in this investigation were (i) adult women aged ≥ 40 years with at least one risk factor to develop CVD, (ii) able to provide written informed consent and possess the ability to communicate fluently in English, and (iii) residing in Montreal, Quebec, Canada. We recruited the eligible participants through a convenience sampling strategy [17] within the clinic setting. Eligibility focused on age and CVD-related risk factors, i.e. obesity (body mass index ≥ 30), smoking, diabetes mellitus, dyslipidemia, and a history of hypertension during pregnancy. These risk factors were confirmed through participant self-report during the recruitment process. Women with a prior diagnosis of cardiovascular disease or those unable to provide informed consent were excluded from the study.

The study was approved by the Institutional Review Board of McGill University, Faculty of Medicine, and Health Sciences (A03-822-21A). All participants were fully informed and gave their consent, ensuring the integrity of our research.

### Data collection

During the period of recruitment, which lasted approximately 5 months, the first author (I.B.) and a trained research assistant approached women in the waiting room to assess their eligibility to participate in the study. If deemed eligible, we introduced the study to them, and they were invited to participate.

Once participants provided their consent, they received a demographic and socio-economic questionnaire, developed by the research team, via LimeSurvey (Supplementary Table S1). The first author (I.B.), who holds a BSc Honours in Cell and Molecular Biology and has training in qualitative research methods, scheduled and conducted the interviews. Interviews lasted approximately 60 minutes and were conducted remotely via Zoom.

We conducted 15 interviews from 11 June 2021 to 26 May 2022. Conversations centered on the understanding of patients regarding CVD, disease prevention, and their needs and preferences for designing Xi-Care. The interview guideline (Supplementary Table S2) was informed by the Decisional Needs Assessment in Populations Framework [18], the NASSS Framework [19], and the System Usability Scale [20].

### Data analysis

We conducted an inductive semantic thematic analysis [21], allowing themes to emerge naturally without predefined categories, following Braun and Clarke’s approach [22]. Interviews were audio-recorded and transcribed verbatim. As all interviews were conducted in English, no translation was required. We coded the transcripts using Dedoose software (version 9.0.107). We refined the coding scheme through iterative comparison to accurately reflect key concepts discussed in the interviews [22]. After 15 interviews, we achieved data saturation, defined as the point at which no new themes emerged from the interviews [23]. Emerging themes were subsequently refined in team meetings.

The research team comprised co-authors who were research trainees (I.B.) and professor researchers (S.A.R., R.G., and C.R.) at the Department of Family Medicine at McGill University. All study participants were aware of this context. Before research team meetings, the first and senior authors met regularly to discuss the findings and resolve any disagreements after the data collection period to ensure that the interpretations of the data were reliable.

To ensure trustworthiness, credibility was supported through iterative team discussions to refine themes; dependability and confirmability were ensured through documentation of analytic decisions and collaborative review of the coding process; transferability was supported by providing detailed descriptions of the study context and participants [24].

## Results

Table 1 presents a detailed summary of the participants’ demographics.

**Table 1:** Sociodemographic characteristics of participants.

Participant	Marital status	Highest level of education	Household income estimated (before tax)	Race/Ethnicity
01	Married	College	More than $62 501	White
02	Single	PhD degree	More than $62 501	White
03	Widowed	MSc degree	More than $62 501	White
04	Married	University degree	$36 001–$4 400	Black
05	Married	MASc in ECE	Equal or less than $25 500	Asian
06	Married	Diploma from Continuing Education at Concordia University	$36 001–$4 400	White
07	Married	University degree	More than $62 501	White
08	Single	Graduate diploma	More than $62 501	White
09	Married	BSc in Nursing	More than $62 501	Black
10	Married	CEGEP & Office Systems Technology Certificate	More than $62 501	White
11	Married	University degree	More than $62 501	White
12	Married	M.A	More than $62 501	White
13	Married	University degree	$44 001–$51 000	Asian
14	Single	Some university	Equal or less than $25 500	Other
15	Married	Certificate in graphic design	More than $62 501	Other

We identified five key themes.

### Theme 1: understanding CVD in a variety of ways

Participants revealed limited awareness and understanding of CVD, often associating it with heart conditions, poor diet, and excessive weight. They noted that many only think of CVD in terms of events like heart attacks, overlooking the broader spectrum, which significantly affects quality of life.

“I don't know about cardiovascular diseases frankly. I’m not familiar with what even they'd be called… what I know is generally about people who have had cardiovascular issues is that they have some kind of a heart condition.”—Participant 3

Meanwhile, a few participants provided insights regarding the unique risks and symptoms of CVD in post-menopausal women. They emphasized the need for tailored awareness initiatives to better recognize and differentiate the signs of disease in women compared to men.

“I think women signs let's say for cardiovascular disease are very different from men. There is some awareness, but I think we, we could emphasize a little bit more so that people can recognize the signs, the different signs versus men and women”—Participant 1

### Theme 2: barriers and challenges to women’s cardiovascular health prevention

Women face multiple barriers in seeking education about CVD prevention. Many participants admitted limited knowledge but showed keen interest in learning more about the disease and preventive measures. They attributed that issue to several deficiencies in women’s cardiovascular health education, misperceptions, and a lack of accessible resources: “Well, first thing comes to mind it's always about men, rarely hear about women”—Participant 7.

Participants reported that they face challenges in primary prevention of CVD, such as the lower visibility of the condition in public health initiatives compared to diseases like breast cancer. “I think there's a lack of it. I'm like I said earlier, we rarely hear about it. For men, it's a different story but for women it's barely out there.”—Participant 7

Moreover, participants stressed the importance of thorough self-reflection to evaluate and adjust their habits while acknowledging the difficulty in prioritizing health and well-being given their diverse obligations: “Prevention is better than cure… For women, it's more challenging, [than] men, because [as] women, we have to work more, and we have more responsibility.”—Participant 05. And: “I think I think we're dealing with so many things on the time we don't put ourselves first.”—Participant 12

### Theme 3. Women taking charge of their cardiovascular well-being

Participants were interested in various approaches to making decisions about their cardiovascular health that included conducting personal search, consulting healthcare experts, and discussing with their social circles. They often combined these approaches, ensuring they were well-informed when speaking with healthcare experts. Despite respecting professional advice, they maintained control over their final decisions: “I don't believe in a doctor making all my decisions because when I go to his office. I expect to have a discussion, not a mandate.”—Participant 04

Moreover, participants highlighted that it is crucial to recognize the role of cultural contexts and literacy levels in these decisions. Participants advocated for collaborative decision-making with physicians, moving beyond a one-sided directive approach. They emphasized patients’ active involvement and the need for doctors to consider their patients’ cultural and dietary preferences to enhance engagement and adherence.

“I really believe that when we go to doctor's office, we're not just there to be told what we have to do. And I believe that the patient should be involved in that decision-making… Simply because we're from different ethnic backgrounds; we do things differently; we eat differently; and therefore, it's important for the doctor or whoever it is to know what these foods are”—Participant 4

### Theme 4. Mixed perspectives regarding the use of AI-enabled technologies for CVD prevention

Many participants were interested in health-related applications, particularly those beneficial to patients and physicians. Participants appreciated the role of modern technology in improving health outcomes. However, they highlighted the importance of accessibility in these AI-enabled technologies: “I think it is important because right now, especially [for] the young generation, and of course… older as well… They are affected by mobile technology”—Participant 5.

As these technologies should aim to raise awareness and encourage proactive health management without being prescriptive, AI-enabled tools have to accommodate various groups based on their age, language, lifestyle, and culture.

“I do like it. I’m very techie …I think if I had an app that would provide me [with] some information and choices. I would still go, and [get] research done…I don’t think I could base my decision on information provided to me in that sense. I have to go back and then and look it up first. I need more than just lay information”—Participant 1

Although some participants described their familiarity with technology like robots and the general concept of AI, they stressed their lack of literacy to comprehend the overall idea of using these technologies: “I never heard of the, what do you call it again. Artificial intelligence system or robot so I am not able to give an answer on that right now.”—Participant 4.

### Theme 5. Range of suggestions for the format and Design of Prospective AI-enabled Technologies

Participants emphasized the need for customization to cater to diverse demographics and individual preferences. A visually appealing application with multilingual support was also highlighted, especially for older women and non-native speakers: “For sure multi-language that's, that's something that is important.”—Participant 01. And: “Colorful appealing. it's it …[has] to be visually appealing. You want to go into it.”—Participant 10

Personalizing features, goals, and risk assessments to align with each user's needs was crucial for user engagement and experience. They also recommended features to monitor performance, provide real-time reminders, and visually represent data using charts. Simplifying the user experience and considering varying digital literacy levels were deemed essential: “The least amount of work that they're going to have to do the better in terms of like the user experience”.—Participant 14.

Participants also highlighted the importance of data collection features for user engagement. The balance between automated data collection and manual input was considered important to reduce user burden. Likewise, it appeared important to integrate comprehensive monitoring of health risks and user behaviors into collaborative efforts with medical professionals and other health monitoring applications.

“If you can automate things… for example, with the Apple health app, then I think that would be really cool because and they would have like an overall overview of their health. That's important… The integrative aspects of it, I think, is really important terms of an app.”—Participant 14

Furthermore, during the interviews, participants gave suggestions about six specific features for AI-enabled technologies like Xi-Care to consider for future developments, as summarized in Table 2.

**Table 2:** Participant suggestions on key features of Xi-care tool.

Feature	Suggestions	Quotes
Educational modules on cardiovascular health	Succinct summaries with hyperlinks to authoritative sources. Multimedia elements like videos, images, and infographics. Integration of periodic reminders with cardiovascular health-related information. Emphasis on quality of life over mortality.	“Especially for those who are not educated, I would say like a video is very helpful, easy to understand.”—Participant 9 “[if] It was a quick message, saying… drinking… X number of litres of water a day is great for your cardiovascular health. That's great… I’ll process that piece of information. But…[am I] going to go read, that I should be drinking water, and I should be this… probably not.”—Participant 12 “I think one of the things that people need to be told is. Look, it's not about whether you live or die. It's a question of how you live before you die.”—Participant 2
Diet recommendations	Introduction of reinforcement mechanisms and challenges or milestones. Video-based culinary courses, recipes, and information about essential nutrients. Constructive approach focusing on foods to improve cardiovascular health rather than restrictions.	“Well, fun challenges are fun awards…[to] keep them [users] motivated to follow these diet recommendations.”—Participant 14 “A video for a cooking with lessons”—Participant 13 “There's a lot of recommendations that I don't want. I'm very selective in terms of what I eat, and there's a lot of stuff that is recommended that I don't eat.”—Participant 03. And: “When you want to change people's eating habits, it's easier to tell them…eat one piece of fruit in the afternoon, as opposed to saying, don't eat a donut in the afternoon… deprivation doesn't get very far.”—Participant 2
Guided personalized exercise	Personalizing exercise routines based on age, physical condition, and preferences. Guidance from experts, such as physiotherapists, for customized workouts.	“If it was a personalized program, I would like that. Okay, [for example] videos, I mean there's so many on YouTube… and there's so many apps … but if they would … take my age … [and] take my condition…personalize exercise would be good.”—Participant 1 “I guess generally when it comes to something like that, I guess I would probably prefer … a physio, or somebody like a professional person who would have spoken to me and assessed me.”—Participant 15
Monitoring and tracking of health data	Tracking health progress: making informed decisions with follow-up health data. Monitoring changes in health over time. Addressing concerns about the addictive nature of monitoring technologies.	“Tracking data is important because…if I start this month, and I… continue to input my data for a long time, so I can understand that improvement or… other negative side… I mean that rate of change so that [it] will help me to understand, like the quality and the progress of my health.”—Participant 5 “I would like to know if there are things to be aware of that I might not know myself because I think we tend to just put things off …I guess the fear is that … it's going to indicate things that aren't, I guess it [monitoring tool] has to be pretty precise.”—Participant 12
Step count (Pedometer)	Predefined goals and challenges. Simplifying regular exercise for individuals with limited access or motivation. Information on health benefits of step counts.	“It could be something that I would possibly adhere to increase and improve my health. but just to tell the steps, without like a goal, to me, it's not something that I look at.”—Participant 1 “It will help me to know. I mean workout is important for us but… I think it's difficult is sometimes.”—Participant 05. And: “It obviously can differ among the different generations. You know the different … life stages. basically, so, I think that would change, depending on the lifecycle state… Maybe for a senior, it would be more important.”—Participant 14
Weight tracking	Combining weight tracking with other features and personalizing it for different users. Avoiding daily tracking to reduce stress. Focusing on sustaining a healthy lifestyle instead of fixating on weight.	“If the weight tracking feature had different options, I guess, for different people, according to… what they decide with their dietitian or doctor, whatever.”– Participant 2 “If an app is something that's meant to be used from day to day. Then I don't think putting the weight in is helpful if the app is going to include … a feature every three, … six or nine months. You know, tracking your overall. I mean I don't think people should be looking at their risk, you know, recalculating their risk you know every week… recalculating the risk might be something that can be done every three months, you know, monitoring the behaviours. As a process that's that can be done more often.”—Participant 2

## Discussion

The findings suggest that, while there is some awareness of CVD among women, it often encompasses a limited view that overlooks the comprehensive range of complications. The disparity between the reality of CVD as the leading cause of mortality among women and their awareness and understanding of disease risk factors and implications is concerning. This gap, emphasized by Roth *et al.* [25], highlights the disconnect in women's recognition of their susceptibility to CVD, in addition to the declining engagement with cardiovascular health among younger women, in contrast to their older counterparts [26, 27]. This demographic discrepancy contributes to an underestimation of the disease as a significant health threat, even though it remains the leading cause of death among women [26, 27].

Moreover, the findings draw attention to the health education barriers experienced by women regarding CVD prevention. It was clear that there is an inadequate focus on CVD in public health initiatives targeting women, where other health concerns like breast cancer often overshadow it. This observation aligns with Berry *et al.* [26] and Sutantri *et al.* [28], who noted an underestimation of CVD risk among women compared to a heightened awareness of breast cancer. The lack of sex-specific health guidelines intensifies this issue, pointing to a significant educational gap in public health [29]. Furthermore, societal expectations and numerous obligations challenge women, making it difficult to balance family and professional responsibilities, which makes prioritizing personal health harder, often leading to neglected self-care and well-being [30].

Our study points to a growing interest in AI-enabled technologies with an emphasis on the need for features that foster an engaging user experience. In fact, there is a broader demand for personalization in health technology, reflecting the essence of feminist intersectionality, which recognizes diverse identities, including differences in race and age [31, 32]. The pursuit of a user-centered design is as important, aiming to diminish biases and boost the efficacy of such technologies for an inclusive user base [33]. Integrating intersectional sex analyses in development of AI-enabled technologies ensures interventions are precisely tailored to distinct population subgroups, respecting the heterogeneity of experiences, particularly for older users who may not have the same level of technological literacy. Engaging diverse stakeholders in the development stages ensures that all users’ viewpoints and requirements, especially those from underrepresented groups, are actively integrated into healthcare solutions.

Our research team has identified important features to be considered in the development of future AI-enabled technologies such as the Xi-Care tool. For instance, a prominent feature of Xi-Care would be the “Educational Modules,” which aims to improve awareness of primary prevention of CVD. These modules are crucial for enhancing women's understanding of diseases, empowering women, and improving health outcomes, aligning with evidence that educational interventions significantly enhance self-efficacy [34]. Likewise, the “Personalized Diet Recommendations” feature would provide practical dietary advice tailored to the user's preferences and lifestyle. By transforming complex nutritional information into engaging video tutorials, this feature would simplify healthy eating, boosting user confidence in their dietary choices [35]. Another significant feature would be the “Customizable Workout Plans,” offering personalized exercise regimens based on the user's health condition, fitness level, and goals, ensuring the workout aligns with their daily routine for sustainable health outcomes [36]. The “Step Count” functionality would emphasize mobility and provides a straightforward method to encourage physical activity, particularly beneficial for users with limited time or access to exercise facilities. Additionally, the “Health Data Monitoring and Tracking” feature would stand out by allowing users to track key health metrics like blood pressure and cholesterol levels, supporting the quantified self-movement and empowering users to make informed health decisions [37]. Although “Weight Tracking” was less popular among participants, it would remain available for those who find it helpful in their health management. Together, these features ensure Xi-Care offers a comprehensive, user-centered approach to cardiovascular health, addressing the diverse needs of women at risk of CVD.

This study represents a pioneering initiative in primary care, focusing on gathering women's perspective on Xi-care, which would be tailored for the primary prevention of CVD in women. It explores the connection between women's health and AI-enabled tools within a primary care setting, substantially reinforcing the study's academic rigor, relevance, and potential impact within primary care and digital health research domains. However, we acknowledge certain limitations such as a focus on a specific subset of women at risk of CVD from one clinic in an urban area, limiting the transferability of the findings. The socio-economic and cultural backgrounds of participants may not correspond to the full array of Xi-Care's user base, potentially skewing the outcomes [12].

## Conclusion

Whereas women's knowledge about CVDs and preventive measures remains an issue in primary healthcare, this study also unveils women's acknowledgement of the lack of sex-specific educational initiatives. Importantly, it also suggests an openness toward AI-enabled technologies that could assist in navigating the primary prevention of CVD among women. Incorporating women's perspectives on cardiovascular health into AI-enabled technologies, such as Xi-Care's features could lead to earlier, sex-sensitive interventions, and personalized initiatives. Engaging patients in the development of AI-enabled technologies like Xi-Care will meet the diverse needs of women at risk of CVD, enhancing the quality of care for women, and aligning with the personalized nature of family medicine.

## Supplementary Material

cmag041_Supplementary_Data

## Data Availability

The data that support the findings of this study are not publicly available due to the need to protect the confidentiality and anonymity of participants. Requests for access to anonymized excerpts or additional information may be considered on a case-by-case basis by contacting the corresponding author, subject to ethical approval and safeguarding of participant privacy.
