# Supplementary material for: The digital heartbeat: a qualitative descriptive study on women's views on preventing cardiovascular disease in primary care
Source: Fam Pract. 2026 Jun 19;43(4):cmag041. doi: 10.1093/fampra/cmag041 (PMC13280644; doi:10.1093/fampra/cmag041)
Supplement: cmag041_Supplementary_Data [file cmag041_supplementary_data.zip › BOUSBIAT_et_al_Suppl._Table_1_24March2024(FV).pdf]

Supplemental Table 1. Pre-Interview Demographic Questions (LimeSurvey)

*What is your marital status?
• Single<br>• Married<br>• Divorced<br>• Widowed
*What is your highest level of education?
*What is your household's estimated yearly income before tax?
• Equal or less than $25,500<br>• $25,501 - $36,000<br>• $36,001 - $44,000<br>• $44,001 - $51,000<br>• $51,001 - $57,000<br>• $57,001 - $62,500<br>• More than $62,501
*How do you identify your race/ethnicity? Please check all that apply.

• Aboriginal (Inuit, Metis, North American Indian)<br>• Arab/West Asian (e.g. Armenian, Egyptian, Iranian, Lebanese, Moroccan)<br>• Asian<br>• Black (e.g. African, Haitian, Jamaican, Somali)<br>• Latin American<br>• Southeast Asian<br>• White (Caucasian)<br>• Other
*Please type your first name and last name below.
