# Supplementary material for: The digital heartbeat: a qualitative descriptive study on women's views on preventing cardiovascular disease in primary care
Source: Fam Pract. 2026 Jun 19;43(4):cmag041. doi: 10.1093/fampra/cmag041 (PMC13280644; doi:10.1093/fampra/cmag041)
Supplement: cmag041_Supplementary_Data [file cmag041_supplementary_data.zip › BOUSBIAT_et_al_Suppl._Table_2_24March2024(FV).pdf]

Supplemental Table 2. Interview questions for women at Increased risk of cardiovascular diseases

**Section I: Needs assessment:** Inquire about participants' current diagnosis and treatment in primary health care.	
How do you think cardiovascular diseases are generally described and understood by the public?<br>Yes/No<br>Comment	i.e., What comes to mind?
Have you ever been informed about your cardiovascular health? How?<br>Yes/No<br>Comment	Follow-up questions:<br>• How are you informed?<br>• What are some questions that you have had about cardiovascular health/diseases, if any?
What are the most frequent and important decisions you face related to your cardiovascular health?	Follow-up questions:<br>• What do you do when faced with making important decisions?<br>• **Note:** focus on if there is an awareness of a decision to be made (i.e. prevention of CVD in system 2)

What is your usual role in making decisions about your cardiovascular health? What do you want your role to be?	Probe role:<br>• Do you usually:<br>a. Make the decisions yourself<br>b. Share the decisions with the doctor<br>c. Your doctor makes the decision for you<br><u>Follow-up question:</u><br>• Who else besides yourself is typically involved in the decision process?
What do you think are some challenges and needs in preventing and managing cardiovascular diseases (from your perspective as a woman at risk of CVD)?	Probes if they need examples for ideas:<br>• Challenges examples: lack of resources (e.g. information, appropriate tools for decision making)<br>• Needs examples: a guide on how to maintain cardiovascular health<br>Note: Probe a bit more, this is a very important question. Ask: “Are there any other needs or challenges you have in this context?”

**Section II: The Xi-Care Tool:** Introduce a technology that aims to assess and prevent cardiovascular diseases to the participants.

Have you ever considered a decision support system to help you in any decisions related to your cardiovascular health?<br>Yes/No<br>Comment	<u>Follow-up questions:</u><br>• If yes, how was your experience?<br>• If yes, what is missing or needs to be considered?<br>• If no, will you be interested in using one?<br>• Is it something you think you would need?<br>Note: decision aids can be used in any context and may not necessarily be collaborative (but they also can be).
What are your thoughts on using digital technology (e.g., mobile apps, AI systems/robots) to make decisions in relation to your cardiovascular health?	Note: explain AI if the question or the concept was not clear.
Questions adapted from NASSS framework.	
How would you like us to design and develop this Xi-Care tool that is useful, helpful and effective for women at risk of CVD (e.g. no risks to users)?	Probe:<br>• What are features would you like to include?<br>Note: You can ask that as a general question and see if they can think of ideas off the top of their heads.<br>If they mention one of the features that we have included on the following slides (i.e. 20 – 25), you can say “ok, how would you rate that on a scale of 1 to 5, one because not at all helpful and 5 being very helpful?”

<table>
<tr><td colspan="2">Questions adapted from the Systems Usability System.</td></tr>
<tr><td>On a scale of one to five, one being not at all helpful and five being very helpful, what features would you find helpful in preventing and managing CVD?
1 (least helpful)
2 (Somewhat unhelpful)
3 (Neither helpful nor unhelpful)
4 (Somewhat helpful)
5 (Very helpful)</td><td>Probe features:
Monitoring tools that track health data over time?
Step-count feature?
Weight tracking feature?
Educational modules on cardiovascular health?
Guided exercise activities? (Personalized exercise)
Diet recommendations?
<u>Follow-up questions:</u>
‘Not helpful’ answers: why this would be not helpful?
‘Helpful’ answers: how would this help you?</td></tr>
<tr><td>Would you like to be able to follow your progress and receive push-notifications through the Xi-Care tool?</td><td><u>Follow-up questions, if yes:</u>
Would you like to receive push-notifications? If so, what about?
How often would you like to receive notifications?
a. Multiple times a day?</td></tr>
</table>

	b. Once at the beginning of every day? At the end?<br>c. Once a week?<br>d. Once a month?<br>e. Customizable?
Questions adapted from NASSS framework.	
How difficult/easy do you think it will be for you to integrate the Xi-Care tool into your daily life?<br>• 1 (Very difficult)<br>• 2 (Somewhat difficult)<br>• 3 (Neither difficult nor easy)<br>• 4 (Somewhat easy)<br>• 5 (Very easy)	<u>Follow-up questions:</u><br>• How often would you see yourself using the Xi-Care tool?<br>• What will facilitate its integration in your daily life activities?<br>• What type of support will you need for this technology; do you need any interventions for learning this technology?
